# Supplementary material for: Female mentors positively contribute to undergraduate STEM research experiences
Source: PLoS One. 2021 Dec 2;16(12):e0260646. doi: 10.1371/journal.pone.0260646 (PMC8638905; doi:10.1371/journal.pone.0260646)
Supplement: S1 Table — (PDF) [file pone.0260646.s001.pdf]

Supplementary Table 1. Summary of sample and response rates.

	**Undergraduates**	**Alumni**
**Males**	139/304	73/180
**Females**	165/304	107/180
**Response Rate**	304/359	180/1163
