# Supplementary material for: Female mentors positively contribute to undergraduate STEM research experiences
Source: PLoS One. 2021 Dec 2;16(12):e0260646. doi: 10.1371/journal.pone.0260646 (PMC8638905; doi:10.1371/journal.pone.0260646)
Supplement: S2 Table — (PDF) [file pone.0260646.s002.pdf]

S2 Table. Summary of responses to gender and mentor selection questions.

	Females (Alumni & Undergraduates)		Males (Alumni & Undergraduate)		p value
	No	Yes	No	Yes	
Did the gender of your mentor influence why you selected them?	255/270	15/270	201/211	10/211	0.837
Do you believe gender should be considered when selecting a mentor?	246/271	25/271	199/212	13/212	0.236
Do you believe males should mentor females and females should mentor males	115/266	151/266	104/204	100/204	0.113
