# Supplementary material for: Female mentors positively contribute to undergraduate STEM research experiences
Source: PLoS One. 2021 Dec 2;16(12):e0260646. doi: 10.1371/journal.pone.0260646 (PMC8638905; doi:10.1371/journal.pone.0260646)
Supplement: S3 Table — (PDF) [file pone.0260646.s003.pdf]

S3 Table. Summary of assignment of mentors based on student gender and mentor gender.

	Female Students		Male Students		p value
	Male mentor	Female mentor	Male mentor	Female mentor	
BIOL 375 Students	38/79	41/79	35/64	29/64	0.198
BIOL 420 Students	35/83	48/83	38/74	36/74	0.128
Undergraduates (BIOL 420 + BIOL 375 students combined)	73/162	89/162	73/138	65/138	0.161
Alumni	70/106	36/106	51/72	21/72	0.271
Alumni + Undergraduates	143/268	125/268	124/210	86/210	0.238
