# Supplementary material for: Female mentors positively contribute to undergraduate STEM research experiences
Source: PLoS One. 2021 Dec 2;16(12):e0260646. doi: 10.1371/journal.pone.0260646 (PMC8638905; doi:10.1371/journal.pone.0260646)
Supplement: S4 Table — (PDF) [file pone.0260646.s004.pdf]

S4 Table. Summary of responses to questions about undergraduate research experience and mentors.

	Alumni			Undergraduates			p value
	No	Maybe	Yes	No	Maybe	Yes	
My undergraduate research experience was positive	1/161	14/161	146/161	2/150	5/150	143/150	0.081
My research mentor was a good role model	8/161	16/161	137/161	1/153	15/153	137/153	0.078
My research mentor was helpful	5/164	16/164	143/164	2/154	16/154	136/154	0.619
My research mentor was understanding	6/160	15/160	139/160	1/153	7/153	145/153	0.042
My research mentor was available to help	4/163	11/163	148/163	3/153	18/153	132/153	0.309
I believe the gender of my mentor contributed to our relationship	111/152	24/152	17/152	100/135	13/135	22/135	0.183
I believe the gender of my mentor contributed to the outcome of my research experience	133/155	10/155	12/155	124/138	5/138	9/138	0.489
I believe my research mentor was biased due to my gender	145/157	7/157	5/157	147/150	0/150	3/150	0.026
I would recommend my research mentor to either gender	13/162	5/162	144/162	3/154	3/154	148/154	0.027
I would recommend my research mentor to females	12/161	8/161	141/161	6/152	6/152	140/152	0.385
I would recommend my research mentor to males	8/161	10/161	143/161	6/152	5/152	141/152	0.425
My undergraduate research prepared me for work in hostile environments	70/134	34/134	30/134	26/128	55/128	47/128	0.001
My undergraduate research prepared me for work for opportunities in my field	19/160	25/160	116/160	6/152	36/152	110/152	0.013
My undergraduate research prepared me for work opportunities due to my gender	91/138	23/138	34/138	77/111	16/111	18/111	0.875
I believe my undergraduate research experience prepared me for disadvantages due to my gender	106/133	18/133	9/133	89/116	18/116	9/116	0.869
I believe my undergraduate research experience prepared me for a career in science	15/164	17/164	132/164	6/152	22/152	124/152	0.119
I believe my undergraduate research	24/127	27/127	76/127	16/94	24/94	54/94	0.757

experience prepared females for a career in science							

	Females (Undergraduates and Alumni)			Males (Undergraduates and Alumni)			p value
	No	Maybe	Yes	No	Maybe	Yes	
My undergraduate research experience was positive	3/176	14/176	159/176	0/135	5/135	130/135	0.096
My research mentor was a good role model	8/179	25/179	146/179	1/135	6/135	128/135	0.001
My research mentor was helpful	6/182	18/182	158/182	1/136	14/136	121/136	0.386
My research mentor was understanding	5/178	15/178	158/178	2/135	7/135	126/135	0.423
My research mentor was available to help	4/181	15/181	162/181	3/135	14/135	118/135	0.869
I believe the gender of my mentor contributed to our relationship	119/169	26/169	24/169	92/118	11/118	15/118	0.277
I believe the gender of my mentor contributed to the outcome of my research experience	149/172	11/172	12/172	108/121	4/121	9/121	0.56
I believe my research mentor was biased due to my gender	165/177	6/177	6/177	127/130	1/130	2/130	0.228
I would recommend my research mentor to either gender	11/181	4/181	166/181	5/135	4/135	126/135	0.632
I would recommend my research mentor to females	11/179	8/179	160/179	7/134	6/134	121/134	0.959
I would recommend my research mentor to males	7/179	8/179	164/179	7/134	7/134	120/134	0.796
My undergraduate research prepared me for work in hostile environments	56/148	48/148	44/148	40/114	41/114	33/114	0.825
My undergraduate research prepared me for work for opportunities in my field	16/176	38/176	122/176	9/136	23/136	104/136	0.381
My undergraduate research prepared me for work opportunities due to my gender	84/141	23/141	34/141	84/108	16/108	8/108	0.001
I believe my undergraduate research experience prepared me for disadvantages due to my gender	106/145	24/145	15/145	89/104	12/104	3/104	0.032
I believe my undergraduate research experience prepared	12/179	22/179	145/179	9/137	17/137	111/137	1

me for a career in science							
I believe my undergraduate research experience prepared females for a career in science	21/151	34/151	96/151	19/70	17/70	34/70	0.042
