# Supplementary material for: Female mentors positively contribute to undergraduate STEM research experiences
Source: PLoS One. 2021 Dec 2;16(12):e0260646. doi: 10.1371/journal.pone.0260646 (PMC8638905; doi:10.1371/journal.pone.0260646)
Supplement: S5 Table — (PDF) [file pone.0260646.s005.pdf]

S5 Table. Summary of responses to questions about gender and research mentor.

	Students with Female Mentors			Students with Males Mentors			p value
	No	Maybe	Yes	No	Maybe	Yes	
I believe the gender of my research mentor contributed to our relationship	72/124	25/124	27/124	139/165	13/165	13/165	<0.001
I believe the gender of my research mentor contributed to the outcome of my research experience	106/126	10/126	10/126	152/169	5/169	12/169	0.162
I believe my research mentor was biased due to my gender.	131/132	0/132	1/132	163/177	7/177	7/177	0.011
I would recommend my research mentor to either gender	3/134	3/134	128/134	13/184	5/184	166/184	0.14
I would recommend my research mentor to females	6/132	5/132	121/132	12/183	9/183	162/183	0.683
I would recommend my research mentor to males	6/132	6/132	120/132	8/183	9/183	166/183	1
I believe my undergraduate research experience prepared me for a career in science	3/135	14/135	118/135	19/183	24/183	140/183	0.008

	Same Gender Pairing (Females with female mentors and males with male mentors)			Different Gender Pairing (Females with male mentors and males with female mentors)			p value
	No	Maybe	Yes	No	Maybe	Yes	
I believe the gender of my research mentor contributed to our relationship	90/146	28/146	28/146	121/143	10/143	12/143	<0.001
I believe the gender of my research mentor contributed to the outcome of my research experience	124/147	12/147	11/147	134/148	3/148	11/148	0.055
I believe my research mentor was biased due to my gender	150/154	1/154	3/154	144/155	6/155	5/155	0.126
I would recommend my research mentor to either gender	6/159	7/159	146/159	10/159	1/159	148/159	0.062
I would recommend my research mentor to females	7/158	9/158	142/158	11/157	5/157	141/157	0.415

I would recommend my research mentor to males	7/158	9/158	142/158	7/157	6/157	144/157	0.764
I believe my undergraduate research experience prepared me for a career in science	10/160	19/160	131/160	12/158	19/158	127/158	0.909
