# Supplementary material for: Female mentors positively contribute to undergraduate STEM research experiences
Source: PLoS One. 2021 Dec 2;16(12):e0260646. doi: 10.1371/journal.pone.0260646 (PMC8638905; doi:10.1371/journal.pone.0260646)
Supplement: S6 Table — (PDF) [file pone.0260646.s006.pdf]

S6 Table. Summary of alumni responses to questions about undergraduate research experience and current positions.

	Female Alumni		Male Alumni		p value
	No	Yes	No	Yes	
Are you in school or currently employed in a science-related field?	15/98	83/98	10/62	52/62	1
Do you feel your undergraduate research experience influenced your current education or employment status?	44/96	52/96	21/62	41/62	0.141
Did your undergraduate research mentor and your undergraduate research project adequately prepare you for your current education or employment status?	29/96	67/96	17/61	44/61	0.858
Do you think that the undergraduate research experience adequately prepared females for a career in science?	17/94	77/94	9/59	50/59	0.825
