# Supplementary material for: Female mentors positively contribute to undergraduate STEM research experiences
Source: PLoS One. 2021 Dec 2;16(12):e0260646. doi: 10.1371/journal.pone.0260646 (PMC8638905; doi:10.1371/journal.pone.0260646)
Supplement: S1 Appendix — (PDF) [file pone.0260646.s007.pdf]

S1 Appendix. Undergraduate research mentoring survey.

Undergraduate Research Mentoring Survey

The following survey is being distributed to obtain information about your undergraduate research experience in the Biology Department at the University of Nebraska at Kearney (UNK). In particular, we are trying to track the role of the undergraduate research project in preparing students for future careers and the role of gender in the mentoring process. Please answer all questions completely. If you have any questions about this survey, please contact Dr. Kim Carlson or Dr. Julie Shaffer in the Biology Department at UNK. All answers are anonymous and we be treated as such. Thank you for your time!

I. Background Information (select only one)

A. What is your gender? O Male O Female

B. What is your age? O 18-20 O 21-30 O 31-40 O 41-50 O 51+

C. What is your ethnic origin?

O Caucasian O Hispanic O African American O Asian O Native American O Other

D. What is the highest year of school completed?

College: O13 O14 O15 O16 Graduate/Professional school: O17 O18 O19 O20 O21 O22 O23+

E. If still in school, what is your current standing?

O Junior O Senior O Master's seeking program O Doctoral seeking program O Other

F. If still in school, what is your current status? OFull-time student OPart-time student

G. What is your current marital status? OSingle OMarried ODivorced, separated, or widowed

H. Do you currently have any children or are expecting a child? OYes ONo

I. If you answered yes to question H, what age ranges are the child/ren (select all that apply)?

O Currently pregnant O 0-1 year O 2-5 years O 6-9 years O 10-13 years O 14-19 years O 20+ years

II. Mentor selection for undergraduate research (select only one for each question)

A. Your undergraduate research mentor is/was: O Male O Female

B. Did the gender of your mentor influence why you selected them? O Yes O No

C. Do you believe gender should be considered when selecting a mentor? O Yes O No

D. Do you believe males should mentor females and females should mentor males? O Yes O No

Why or why not?

E. Why did you select your research mentor?

BIOL 375 STUDENTS STOP HERE.

III. Undergraduate research experience

	No	Maybe	Yes	Not applicable or unsure
A. My undergraduate research experience was positive	O 0	O 1	O 2	O 3
B. My research mentor was a good role model	O 0	O 1	O 2	O 3
C. My research mentor was helpful	O 0	O 1	O 2	O 3
D. My research mentor was understanding	O 0	O 1	O 2	O 3
E. My research mentor was available to help	O 0	O 1	O 2	O 3
F. I believe the gender of my research mentor contributed to our relationship.	O 0	O 1	O 2	O 3

G. I believe the gender of my research mentor contributed to the outcome of my research experience.	O 0	O1	O2	O3
H. I believe my research mentor was biased due to my gender.	O 0	O1	O2	O3
I. I would recommend my research mentor to either gender	O 0	O1	O2	O3
J. I would recommend my research mentor to females	O 0	O1	O2	O3
K. I would recommend my research mentor to males	O 0	O1	O2	O3
L. I believe my undergraduate research experience prepared me for hostile work environments.	O 0	O1	O2	O3
M. I believe my undergraduate research experience prepared me for opportunities in my field.	O 0	O1	O2	O3
N. I believe my undergraduate research experience prepared me for opportunities due to my gender.	O 0	O1	O2	O3
O. I believe my undergraduate research experience prepared me for disadvantages due to my gender.	O 0	O1	O2	O3
P. I believe my undergraduate research experience prepared **me** for a career in science.	O 0	O1	O2	O3
Q. I believe my undergraduate research experience prepared **females** for a career in science.	O 0	O1	O2	O3

R. What were the problems with your undergraduate research experience in relationship to your mentor?

S. Do you feel you are adequately prepared for a career in science?

IV. **POST-GRADUATE QUESTIONS** – **Only** complete this section if you have completed your undergraduate research experience (BIOL 420) **AND** have graduated from UNK.

A. How long ago did you perform your undergraduate research project?

O 0-6 months O 7-12 months O 2-3 years O 4-5 years O 6+ years

B. Are you in school or currently employed in a science-related field? O Yes O No

C. Do you feel your undergraduate research experience influenced your current education or employment status? ? O Yes O No

D. What is the gender of your current supervisor or mentor? O Male O Female

E. Did the gender of your undergraduate research mentor influence your choice of your current supervisor or mentor? O Yes O No O I did not have a choice

F. Did you undergraduate research mentor and your undergraduate research project adequately prepare you for your current education or employment status? O Yes O No

G. Do you think that the undergraduate research experience adequately prepared **females** for a career in science? O Yes O No

H. What should be done to more adequately prepare **females** for a career in science?

I. What do you suggest should have been done to adequately prepare all students for a career in science?

J. Any other comments or suggestions?
